# A Cost–Consequence Analysis of Preemptive *SLCO1B1* Testing for Statin Myopathy Risk Compared to Usual Care

**DOI:** 10.3390/jpm11111123

**Published:** 2021-10-31

**Authors:** Charles A. Brunette, Olivia M. Dong, Jason L. Vassy, Morgan E. Danowski, Nicholas Alexander, Ashley A. Antwi, Kurt D. Christensen

**Affiliations:** 1 Veterans Affairs Boston Healthcare System, Boston, MA 02130, USA; jvassy@partners.org (J.L.V.); morgan.danowski@va.gov (M.E.D.); nlsandor@bu.edu (N.A.); ashley.antwi@va.gov (A.A.A.); 2 Duke Center for Applied Genomics & Precision Medicine, Department of Medicine, Duke University School of Medicine, Durham, NC 27705, USA; odong@rti.org; 3 Durham VA Health Care System, Durham, NC 27705, USA; 4 Department of Medicine, Harvard Medical School, Boston, MA 02215, USA; kurt_christensen@harvardpilgrim.org; 5 Division of General Internal Medicine and Primary Care, Brigham and Women’s Hospital, Boston, MA 02115, USA; 6 Population Precision Health, Ariadne Labs, Boston, MA 02215, USA; 7 Department of Population Medicine, Harvard Pilgrim Health Care Institute, Boston, MA 02215, USA

**Keywords:** *SLCO1B1*, statin-associated muscle symptoms, pharmacogenetics, cost–consequence analysis, cardiovascular disease, precision medicine

## Abstract

There is a well-validated association between *SLCO1B1* (rs4149056) and statin-associated muscle symptoms (SAMS). Preemptive *SLCO1B1* pharmacogenetic (PGx) testing may diminish the incidence of SAMS by identifying individuals with increased genetic risk before statin initiation. Despite its potential clinical application, the cost implications of *SLCO1B1* testing are largely unknown. We conducted a cost–consequence analysis of preemptive *SLCO1B1* testing (PGx+) versus usual care (PGx−) among Veteran patients enrolled in the Integrating Pharmacogenetics in Clinical Care (I-PICC) Study. The assessment was conducted using a health system perspective and 12-month time horizon. Incremental costs of *SLCO1B1* testing and downstream medical care were estimated using data from the U.S. Department of Veterans Affairs’ Managerial Cost Accounting System. A decision analytic model was also developed to model 1-month cost and SAMS-related outcomes in a hypothetical cohort of 10,000 Veteran patients, where all patients were initiated on simvastatin. Over 12 months, 13.5% of PGx+ (26/193) and 11.2% of PGx− (24/215) participants in the I-PICC Study were prescribed Clinical Pharmacogenetics Implementation Consortium (CPIC) guideline-concordant statins (Δ2.9%, 95% CI −4.0% to 10.0%). Differences in mean per-patient costs for lipid therapy prescriptions, including statins, for PGx+ compared to PGx− participants were not statistically significant (Δ USD 9.53, 95% CI −0.86 to 22.80 USD). Differences in per-patient costs attributable to the intervention, including PGx testing, lipid-lowering prescriptions, SAMS, laboratory and imaging expenses, and primary care and cardiology services, were also non-significant (Δ− USD 1004, 95% CI −2684 to 1009 USD). In the hypothetical cohort, *SLCO1B1*-informed statin therapy averted 109 myalgias and 3 myopathies at 1-month follow up. Fewer statin discontinuations (78 vs. 109) were also observed, but the *SLCO1B1* testing strategy was 96 USD more costly per patient compared to no testing (124 vs. 28 USD). The implementation of *SLCO1B1* testing resulted in small, non-significant increases in the proportion of patients receiving CPIC-concordant statin prescriptions within a real-world primary care context, diminished the incidence of SAMS, and reduced statin discontinuations in a hypothetical cohort of 10,000 patients. Despite these effects, *SLCO1B1* testing administered as a standalone test did not result in lower per-patient health care costs at 1 month or over 1 year of treatment. The inclusion of *SLCO1B1*, among other well-validated pharmacogenes, into preemptive panel-based testing strategies may provide a better balance of clinical benefit and cost.

## 1. Introduction

Pharmacogenomic (PGx) testing leverages a patient’s genetic information to guide medication prescribing. Over the last decade, PGx testing has become an increasingly important and continuously expanding feature of personalized clinical medicine [1,2]. At present, the Pharmacogenomics Knowledge Base (PharmGKB) includes over 160 clinical guidelines and nearly 800 medication label annotations describing potential drug–gene effects backed by medical agencies globally [3]. Although many institutions are integrating PGx testing into their clinical care, others remain reluctant to do so given a dearth of rigorous evidence about clinical and cost-related outcomes [4,5].

Of the growing list of actionable drug–gene associations, one of the most well-validated is the interaction between solute carrier organic anion transporter family member 1B1 *(SLCO1B1)* and statin-associated muscle symptoms (SAMS) [6,7,8]. The evidence for this relationship is strongest for simvastatin but may be variably present for other types of statin medications and within specific ancestries with minor C risk alleles present in up to 20% of some populations [8,9,10,11]. Of carriers, greater than 60% of statin myopathy cases may be attributable to the C variant [6]. Moreover, the presence of C alleles (TC or CC) within the *SLCO1B1* genotype at rs4149056 confers an approximate 3-fold increased risk of clinically adjudicated myopathy independent of non-genetic risk factors [12]. Chanfreau-Coffinier et al. [13] project that nearly 2 million U.S. military Veteran patients may harbor such potentially actionable *SLCO1B1* variants across the Veterans Health Administration (VHA). 

Statin therapy is a common and effective approach for reducing death and major cardiovascular disease (CVD) events across nearly all ages and risk profiles [14,15]. However, approximately 20% of statin users report statin-related side effects, with the most commonly reported issue involving muscle pain or weakness [16,17,18]. The overall benefit of statin usage appears to outweigh its immediate harms, including myalgia, myopathy, and rare cases of rhabdomyolysis, as the most deleterious consequence of statin side effects may be patients’ non-adherence or discontinuation of an effective cholesterol-lowering medication [19,20]. Patients who experience statin-related side effects are less likely to satisfactorily reduce their low-density lipoprotein cholesterol (LDL-C) levels, have increased risk for CVD events, have less trust in their doctors, and incur greater health care costs [18,21]. While knowledge of *SLCO1B1* genotype alone does not guarantee that patients taking statins will be free from muscle-related side effects, it does present an opportunity to more accurately identify individuals at increased risk and provide reassurance to those who are not [22]. Recent randomized trials returning *SLCO1B1* genotype information for statin therapy specifically have observed short-term improvements in statin reinitiations and reduced LDL-C [23] and have demonstrated an absence of negative effects on CVD prevention and potential aversion to simvastatin by prescribing physicians for patients at increased genetic risk of SAMS in a primary care setting [24]. 

Evidence to inform decision makers of the costs of *SLCO1B1* testing, and preemptive PGx testing more generally, is also limited [4,22,25]. Only a handful of studies have focused attention on the economic consequences of statin-related PGx-guided prescribing, including both *SLCO1B1* and other variants [26,27,28,29,30,31], with rare emphasis on the cost-related effects of preemptive PGx testing of any kind [32]. By and large, PGx testing related to statins, either as a single test or as part of a panel, appears to be cost effective, but this has only been observed when modeled in specific clinical populations (i.e., acute coronary syndrome) or over long-term timeframes (i.e., lifetimes) [30,31,32]. Additional information is needed to better understand the costs and consequences of standalone preemptive *SLCO1B1* testing over more practical timeframes and general settings, such as primary care. Here, we seek to further elucidate the existing literature by describing the economic costs and consequences of the Integrating Pharmacogenetics in Clinical Care (I-PICC) Study (ClinicalTrials.gov NCT02871934). The I-PICC Study was a pragmatic randomized trial of preemptive *SLCO1B1* testing for statin therapy in primary care and women’s health settings across the Veterans Affairs Boston Healthcare System (VABHS). This report is intended to provide detailed insight of the observed costs and outcomes of administering *SLCO1B1* testing within a real-world clinical setting. To complement the trial data, we additionally developed a decision analytic model to assess standalone *SLCO1B1* testing in a hypothetical cohort of 10,000 Veteran patients. 

## 2. Materials and Methods

### 2.1. I-PICC Study Overview

The I-PICC Study was a pragmatic randomized clinical trial that compared the delivery of *SLCO1B1* (rs4149056) test results to primary care providers versus the treatment as usual. The I-PICC Study was approved by the VABHS Institutional Review Board, and all participants provided informed consent. Detailed descriptions of the study protocol, the pragmatic elements of study design and recruitment and enrollment metrics, and the primary outcomes of the study have been previously reported [24,33,34]. In brief, study participants included primary care and women’s health providers (*n* = 47) and their patients (*n* = 408) across eight sites of the VABHS. VABHS is a large, integrated health system serving military veterans from the Boston metropolitan and surrounding areas of eastern Massachusetts, USA. Patients were eligible for the study if they were between the ages of 40 and 75, were statin naive at enrollment, met at least one criterion for elevated CVD risk per the American College of Cardiology/American Heart Association (ACC/AHA) guidelines [35], had received care at VABHS for a minimum of six months, and were receiving clinical care from an enrolled provider. Using an existing clinical specimen collected during routine care, patients were enrolled into the study by their provider’s signing of an order for *SLCO1B1* testing. Patients were randomized at the point of care to have their providers receive the PGx testing results through the electronic health record (EHR) either immediately (PGx+, *n* = 193) or after 12 months (PGx−, *n* = 215). All patients were followed for one year. All clinical and cost-related outcomes were collected observationally through the EHR and administrative databases. Study recruitment began in December 2016 and all patients completed enrollment as of July 2019. 

### 2.2. Cost–Consequence Analysis 

#### Overall Approach

We conducted a cost–consequence analysis alongside the I-PICC Study randomized trial. The premise of a cost–consequence analysis is to ascertain the direct and indirect costs and outcomes related to an intervention and its alternatives [36,37,38]. From the health system perspective, and using published guidelines from the International Society for Pharmacoeconomic and Outcomes Research (ISPOR) [39] and the Second Panel on Cost-Effectiveness in Health and Medicine [40,41], we assessed total inpatient, outpatient, and intervention-specific costs and outcomes associated with the integration of *SLCO1B1* testing compared to the treatment as usual within primary care clinics of the VABHS. Observed costs and consequences were evaluated over the 12-month study timeframe and modeled within a hypothetical cohort of 10,000 veterans over a 1-month period. All observed costs were adjusted to 2020 USD rates using the U.S. Bureau of Labor Statistics Consumer Price Index for all urban consumers (CPI-U) [42,43]. We adhered to the recommendations of the Consolidated Health Economic Reporting Standards (CHEERS) [44] for the presentation of this work (Supplementary Materials).

### 2.3. VHA Clinical, Service Utilization, and Cost Data 

Per best practice recommendations, outcomes associated with health care utilization included a combination of high-cost resources as well as services expected to differ between randomization arms [39]. Participant-level economic data and health-related outcome data were derived from the EHR and VHA Corporate Data Warehouse (CDW) [45]. The CDW is a repository of VHA administrative and clinical data from the nationally deployed EHR (the Computerized Patient Record System, CPRS). Structured data, including demographic information, procedures and diagnoses, prescriptions for statins and other lipid medications, and provider-documented SAMS, were ascertained. Data associated with health care utilization and costs were obtained from the Managerial Cost Accounting System (MCA) National Data Extracts, housed within the CDW [46,47,48]. The MCA extracts use an activity-based cost accounting method for all individual-level inpatient and outpatient encounters that occur at VHA medical facilities. The system provides precise cost estimates for direct costs, such as provider time and supplies attributable to direct patient care, and indirect costs, including administrative overhead for clinical space and other operational expenses. These expenses reflect costs to the VHA health care system and do not account for costs to the patient nor reimbursements from additional payers. 

Twelve-month health care utilization was characterized using total inpatient stays, mean length of stay, and outpatient encounter days that occurred at VHA medical facilities. Total outpatient encounters included cost and no-cost in-person and remote interactions with VHA providers and services. Health care costs included both high-cost inpatient physical health stays as well as outpatient medical costs. Inpatient encounters were included in the derivation of total costs if they were associated with non-mental health-related treatment. Outpatient utilization and costs were extracted based on clinic stop codes for primary care, cardiology, and ancillary costs related to statin and other lipid medications, laboratory and imaging expenses, and costs for other general medical care (Supplementary Tables S1 and S2). Costs related to SAMS diagnosis or treatment were obtained using Current Procedural Terminology (CPT) and International Classification of Disease (ICD) codes for patients with a statin prescription and provider-documented SAMS only (Supplementary Table S3). Potential SAMS costs were ascertained subsequent to the date of provider documentation of SAMS in the medical record.

### 2.4. Statistical Analysis

Analyses of data from I-PICC Study participants focused on directly attributable services, defined as service costs considered immediately applicable to the study intervention. These services encompassed the cost of primary care and cardiology services, PGx testing, the cost of any lipid therapy prescription, and the potential costs of provider-documented SAMS including imaging and laboratory expenses. Additional analyses considered all observed health care utilization, as noted above.

Data were analyzed using an intention-to-treat approach, including all participants who underwent randomization. Participants were randomized to the study intervention or treatment as usual arms via a two-level (provider and patient) pseudo-cluster randomization procedure [33,49]. To account for clustering at the provider level, we used generalized estimating equations (GEEs) [50,51,52,53,54,55] to estimate between-arm mean differences with cluster bootstrapped 95% confidence intervals [56,57,58] (1000 samples) for 12-month clinical outcomes, utilization, and costs. Statistical models were specified using applicable distributional assumptions (e.g., gamma for right-skewed cost data) and link functions (e.g., ‘log’) with an exchangeable correlation structure. Mean differences, confidence intervals, and *p*-values are presented after adjustment for the correlated data. The analysis of study data was conducted in R (v4.0.2) [59]. GEE models were fit using geepack (v1.3.2) [60]. Accompanying model-based (e.g., SAMS) and cluster bootstrapped confidence intervals were calculated using gee (v4.13-20) [61] and rsample (v0.0.8) [62], respectively. Additional details regarding our primary and supplemental analyses are described in the Supplementary Methods.

### 2.5. Scenario and Sensitivity Analyses

Scenario and sensitivity analyses were conducted to examine the robustness of our observed outcomes. We applied different analytic assumptions to determine how downstream costs changed as a result of including only directly attributable costs [39,63] and by varying the costs of PGx testing and costs of statins. PGx testing cost was varied by ±25% of the actual test cost (99 USD) as well as assessed at no cost to mimic a fully preemptive testing scenario where PGx results were already available. We varied individual statin-related costs from 50% to 200% of observed statin costs. We also assessed cost categories amongst statin users only as well as for statin users carrying at least one risk-enhancing C allele (TC or CC genotype at rs4149056) to capture cost differences associated specifically with actionable results. Parameter and cluster bootstrapped 95% confidence intervals for our scenario and sensitivity analyses were estimated using 1000 samples.

### 2.6. Projected Cost and Health Outcomes 

**Figure 1:**
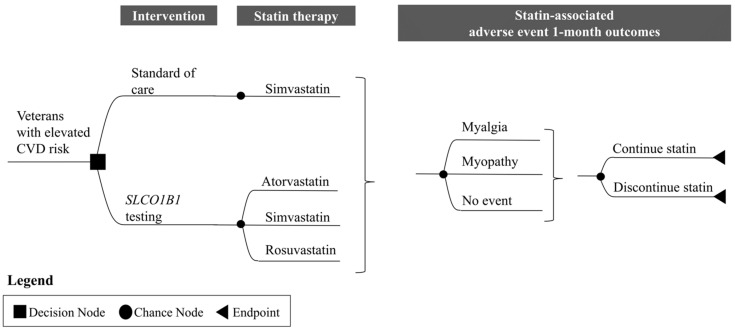
**Model structure for 1-month statin therapy and statin-associated adverse events. (adapted from Dong et al., *Value Health*, 2020 [30]).** A total of 10,000 simulated veterans with elevated cardiovascular disease risk are entered into the decision analytic model and are assigned to standard of care (no testing) and to *SLCO1B1* testing. Statins can be offered and initiated in both testing strategies. Statin therapy options include atorvastatin, simvastatin, and rosuvastatin. After one month of being on statin therapy, the presence of a myalgia and myopathy may occur, which may lead to discontinuation of the statin. Abbreviations: CVD, cardiovascular disease.

A decision analytic model using a decision tree was developed (Oracle Crystal Ball: Redwood City, CA; Microsoft Excel: Redmond, WA) to simulate 1-month projected cost and SAMS-related outcomes from the VHA’s perspective of implementing preemptive *SLCO1B1* testing compared to no testing in a closed cohort of 10,000 veterans with elevated CVD risk who were initiated on simvastatin therapy. The goal of this model was to estimate the maximum potential benefits of *SLCO1B1* testing (best-case scenario). Modeling was largely informed by a previously published cost-effectiveness analysis [30]. Figure 1 displays the structure of the model to compare preemptive *SLCO1B1* testing with standard of care. The baseline statin therapy prescription breakdown was as follows: 100% simvastatin, 0% atorvastatin, and 0% rosuvastatin. For the no testing strategy, changes to initial statin therapy assignment were not completed. Statin-related adverse outcomes at 1 month included both *SLCO1B1*- and non-*SLCO1B1*-related myalgia and myopathy. For the *SLCO1B1* testing strategy, veterans with C alleles and initially prescribed simvastatin were switched before medication initiation to an alternative statin therapy (50% to atorvastatin, 50% to rosuvastatin) to avoid *SLCO1B1*-induced myopathy and myalgia. Rates of statin discontinuation for any reason, among individuals who experienced myalgia or myopathy, were 27% and 40%, respectively [30]. A 1-month timeframe was selected given that most SAMS cases occur within a few weeks after statin initiation [64,65].

The best-case scenario was completed using a point estimate for all input parameters. To provide insight about how uncertainty surrounding input parameters affected model estimates, we conducted probabilistic sensitivity analysis using 1000 Monte Carlo simulations where we sampled input parameters from their underlying distributions, using data from the literature and the I-PICC Study. Beta distributions were used to parameterize 1-month myalgia and myopathy outcomes; triangular distributions were used to parameterize all other inputs. Costs were adjusted to 2020 USD using the methods described above. All model inputs are described in Supplementary Table S4. 

## 3. Results

### 3.1. I-PICC Study Participant Characteristics

A total of 39 providers enrolled 408 patients into the I-PICC Study (median 7, range 1–61). Participants were predominantly male (93.9%) and had a mean age of 64.1 years (Table 1). Of participants who identified with a race or ethnicity, 13.7% self-identified as non-white and 2.0% self-identified as Hispanic. Just over one-third (33.6%) of the sample were considered current smokers. In terms of ACC/AHA risk criteria at baseline, about one-quarter of all participants had prior diagnoses for CVD (24.0%) and diabetes (24.0%), respectively. Only 2.7% of participants had an LDL-C greater than 190 mg/dL. Ninety percent (90.0%) of all participants presented with baseline ACC/AHA risk scores greater than or equal to the 7.5% threshold recommended for statin therapy. Participant characteristics across treatment arms were generally comparable, but a slightly lower proportion of C variant carriers were observed in the PGx+ arm (23.3% vs. 34.9%). Overall, 29.4% of the total sample were C variant carriers, similar to previous projections (25.6%) within the VHA patient population [13].

### 3.2. Clinical Outcomes in I-PICC Study Cohort

There were no significant differences across treatment arms (PGx+ minus PGx−) for lipid prescriptions, provider-documented SAMS, or statin discontinuations (Table 2). Over 12 months, a slightly higher proportion of PGx+ participants were prescribed both Clinical Pharmacogenetics Implementation Consortium (CPIC) guideline-concordant statins (Δ2.9%, 95% CI −4.2% to 9.0%, *p* = 0.34) and non-statin lipid therapies (Δ1.7%, 95% CI −2.0% to 6.0%, *p* = 0.42). Of the total observed statin prescriptions, most were for atorvastatin (76.0%), followed by simvastatin (18.0%), and rosuvastatin (6.0%). Prescriptions for simvastatin trended higher when the *SLCO1B1* genotype was known (Δ17.1%, 95% CI −0.5% to 34.8%, *p* = 0.10), particularly for individuals without a reduced function C allele [24]. Of statin users (*n* = 50), a slightly lower proportion of participants in the treatment arm were observed as having provider-documented SAMS within the EHR (2 vs. 3, Δ−5.5%, 95% CI −22.6% to 11.7%, *p* = 0.53). Only one incident of SAMS was observed related to simvastatin (20 mg), which occurred in a participant with a normal genotype (T/T) in the treatment arm. A slightly lower proportion of participants in the treatment arm were also observed to have discontinued a statin compared to the control group (3 vs. 4, Δ−3.7%, 95% CI −23.2% to 15.8%, *p* = 0.71). Three of the total discontinuations (43%) were associated with patient-reported muscle symptoms, with one such discontinuation in the PGx+ group and two in the PGx- group. CVD-related events were not collected as part of the trial.

### 3.3. Health Care Service Utilization and Costs in I-PICC Study Cohort

There were no significant differences in 12-month VA service utilization between the arms for total inpatient stays (Δ−0.1, 95% CI −0.3 to 0.2, *p* = 0.69), the mean length of stay (Δ0.3 days, 95% CI −5.0 to 4.9, *p* = 0.89), or total outpatient encounters (Δ1.2, 95% CI −4.1 to 6.7, *p* = 0.65) (Table 2). There were slightly fewer outpatient visits for primary care (Δ−0.5, 95% CI −3.8 to 0.4, *p* = 0.66) and cardiology (Δ−0.2, 95% CI −0.6 to 0.1, *p* = 0.13) services in the PGx+ group specifically, but neither difference was statistically significant. Overall, healthcare utilization in this study was consistent with prior assessments of health care service usage within VHA [66,67,68] and for outcomes such as outpatient primary care and cardiology visits and inpatient stays within community settings [69,70].

Differences in 12-month attributable costs between arms, including PGx testing (99 USD) and downstream costs related to lipid-lowering prescriptions, SAMS, imaging and laboratory expenses, and primary care and cardiology services were not statistically significant (Δ−1004 USD, 95% CI −2684 to 1009 USD, *p* = 0.28). On average, 12-month statin and SAMS costs among PGx+ participants were about 2.07 USD (95% CI −1.99 to 6.22 USD, *p* = 0.29) and 2.76 USD (95% CI 0.06 to 5.47 USD, *p* = 0.05) higher than the control group, respectively. Twelve-month average costs for all lipid therapy prescriptions were higher in the PGx+ compared to PGx- group, but the difference was not statistically significant (Δ9.53 USD, 95% CI −1.38 to 22.58 USD, *p* = 0.14) and was further attenuated when accounting for utilization and prior 12-month covariates (range Δ 1.34 to 6.68 USD) (Supplementary Table S5). The intervention arm had lower average total costs for other outpatient services (−544 USD, 95% CI −3314 to 3292 USD, *p* = 0.72) and higher average total costs for inpatient care (880 USD, 95% CI −4142 to 6618 USD, *p* = 0.73) over 12 months, but neither was significantly different from the usual care arm. Differences in the total 12-month mean costs between arms were also non-significant (Δ−52 USD, 95% CI −6660 to 8475 USD, *p* = 0.99). Additional analyses including health care utilization and prior 12-month costs as model covariates demonstrated lower trending costs for directly attributable costs (range Δ – 116 to −725 USD) and total costs (range Δ −1045 to −3150 USD) for the PGx+ group, but overall differences between the arms remained non-significant, particularly when considering multiple comparisons (Supplementary Table S5).

### 3.4. Projected Cost and Health Outcomes in Hypothetical Veteran Patient Cohort

**Figure 2:**
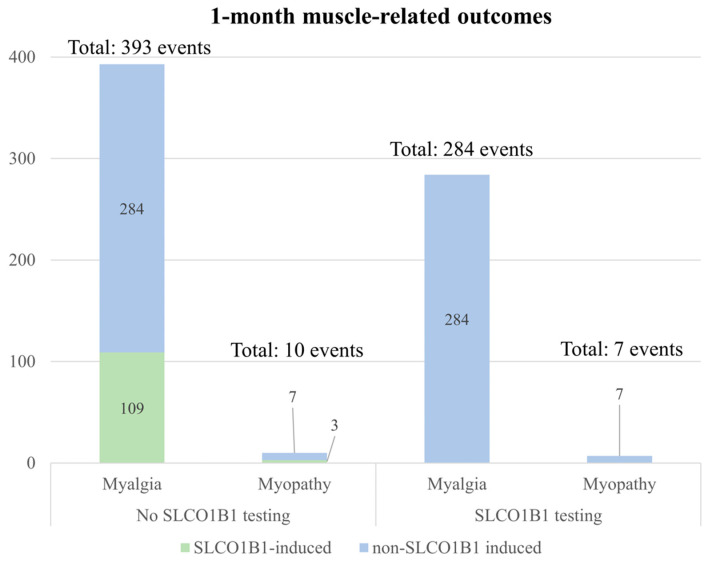
**Estimated event outcomes from modeling the best-case scenario.** The frequency of 1-month muscle-related outcomes for *SLCO1B1* testing and no testing strategies in a hypothetical cohort of 10,000 veteran patients. Outcomes included myalgia and myopathy (*SLCO1B1*-induced and non-*SLCO1B1*-induced outcomes).

**Figure 3:**
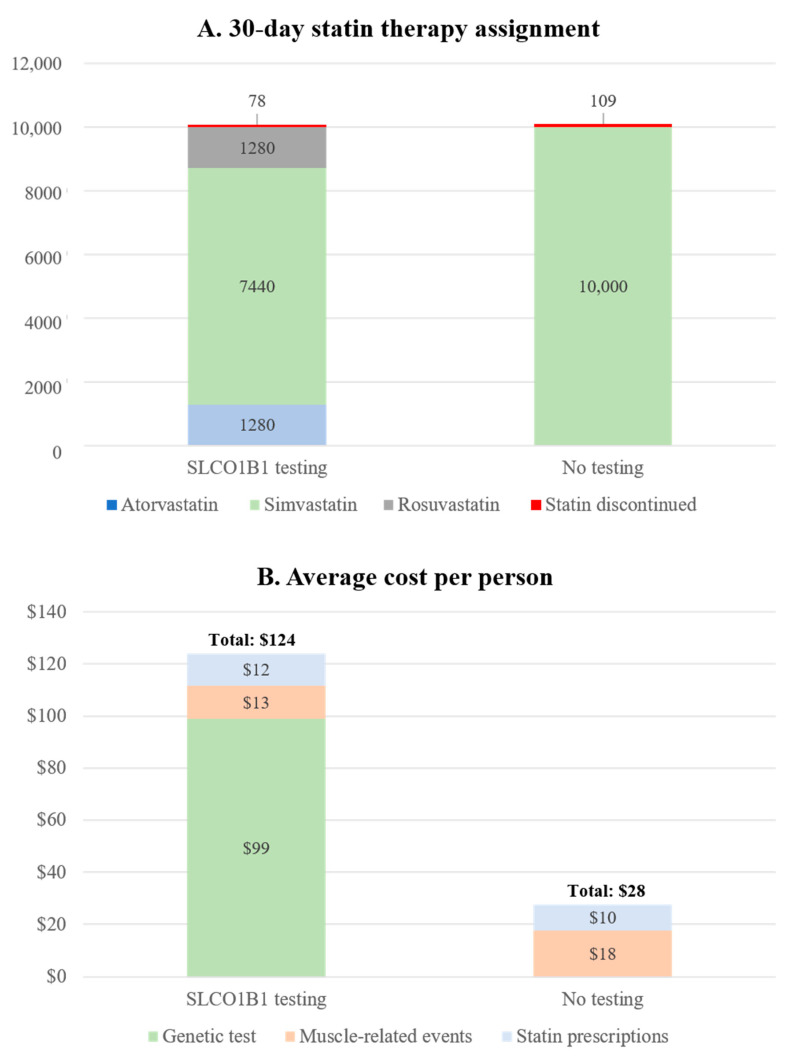
**(A) Statin therapy assignments and (B) average cost per person at 30 days from modeling the best-case scenario.** (**A**) The 30-day statin therapy assignments in a cohort of 10,000 veterans in the *SLCO1B1* testing and no testing strategies. Possible statin therapies include atorvastatin, simvastatin, rosuvastatin, or statin discontinuations. Statin discontinuation is caused by statin-related myopathies and myalgias. (**B**) The average cost per person and cost sources for *SLCO1B1* testing and no testing. Cost categories included genetic testing, muscle-related events, and statin prescriptions. Costs are reported in 2020 USD.

Of the 10,000 veteran patients in the hypothetical cohort who were initially prescribed simvastatin, the best-case scenario for *SLCO1B1* testing projected that 2560 would carry *SLCO1B1* variants and would be switched to an alternative statin therapy prior to initiation. *SLCO1B1* variant carriers in the no testing strategy continued on simvastatin. These *SLCO1B1*-informed statin therapy switches would avert 109 (95% CI 94 to 141) and 3 (95% CI 2 to 5) *SLCO1B1*-induced myalgias and myopathies, respectively (Figure 2, Supplementary Table S6). Fewer statin discontinuations would also be observed for those who would receive *SLCO1B1* results compared to those who would not (78 vs. 109). The *SLCO1B1* testing strategy would be 96 USD more costly per patient compared to the no testing strategy (124 vs. 28 USD) at 1 month. The higher average cost per person in the *SLCO1B1* testing strategy would be mostly driven by the cost of *SLCO1B1* testing (Figure 3).

### 3.5. Scenario and Sensitivity Analyses for I-PICC Study Cohort

A range of costs were considered potentially impacted by the PGx testing intervention. We assessed costs spanning from those immediately associated with the intervention (PGx testing, statins, and SAMS) through broader downstream costs including primary care, cardiology, laboratory, and imaging expenses (Table 3). Higher immediate expenses for PGx testing, statins, and SAMS (Δ104 USD, 95% CI 97 to 112 USD, *p* = 0.001) and for PGx testing, all lipid therapies, and SAMS (Δ110 USD, 95% CI 98 to 123 USD, *p* < 0.001) were observed in the intervention arm over 12 months. Nearly 90% of these differences were driven by the cost of the PGx test (99 USD). Overall, between-arm cost differences trended lower for the intervention arm as broader expense categories were assessed, but these differences lost statistical significance once costs were included beyond immediate expenses. These between-arm trends also held when assessed amongst statin users and non-users independently (Supplementary Table S7) as well as within ACC/AHA high-risk subpopulations (Supplementary Table S8).

One-way variation in the cost of *SLCO1B1* testing (no cost; ±25%) and statin cost (50–200%) had minimal effect on observed mean cost differences between treatment arms (range Δ −102 to 26 USD from base case) (Table 4). Average 12-month immediate costs (lipid therapy prescriptions and SAMS-related costs) were approximately 12.97 USD (95% CI 1.34 to 25.09 USD, *p* = 0.039) higher in the intervention arm when considering a fully preemptive, no cost PGx testing scenario. However, 12-month cost differences between arms under the no cost scenario lost significance as cost categories were expanded. Assessment of costs among the subsample of statin users with the TC or CC genotype only followed similar trends as other analyses, with the intervention demonstrating a statistically significant impact on more immediate expenses compared to total costs over the 12-month time horizon. Of note, statin users in the intervention arm with a TC or CC genotype (*n* = 19) incurred lower immediate costs (Δ74 USD, 95% CI 29 to 119 USD, *p* = 0.001) compared to statin users with a normal function TT genotype (*n* = 31) (Δ121 USD, 95% CI 53 to 190 USD, *p* < 0.001). These between-arm differences stratified by genotype (TC or CC versus TT) remained consistent when considering a fully preemptive, no cost PGx testing scenario (TC or CC: Δ−9.94 USD, 95% CI −29.80 to 9.98 USD, *p* = 0.328; TT: Δ25 USD, 95% CI −33 to 82 USD, *p* = 0.396). Overall, trends were consistent across different scenarios and cost structures, ranging from 12-month immediately attributable costs to 12-month total costs. The most influential factor related to cost impact over the 12-month observation period in I-PICC Study participants was the variation in the cost of PGx testing.

### 3.6. Scenario and Sensitivity Analysis for Projected Cost and Health Outcomes in Hypothetical Veteran Patient Cohort

Results from the probabilistic sensitivity analysis estimated that as few as 332 (3.3% event rate) to as many as 481 (4.8% event rate) muscle-related events would be observed across our hypothetical cohort of 10,000 veteran patients 1 month after statin initiation without *SLCO1B1* testing (Supplementary Table S6). Further, the analysis estimated that as few as 96 to as many as 146 incident cases of SAMS (range 22.3% to 38.2% of total SAMS cases) could be averted by implementing an *SLCO1B1* testing strategy within our hypothetical patient cohort. Estimated costs to the health system per muscle-related event averted at 1-month ranged from 6575 to 10,000 USD.

## 4. Discussion

We leveraged trial data from the I-PICC Study and developed a decision analytic model to assess the costs and consequences of administering a preemptive *SLCO1B1* testing strategy within the primary care setting of a large, integrated health system. Our findings suggest that, while *SLCO1B1* testing may provide some small, non-statistically significant, clinical benefits in the form of increased lipid therapy prescriptions, fewer statin discontinuations, and fewer SAMS cases, administration as a standalone test would be unlikely to reduce costs to the health system over the short term. Overall mean per-patient costs, including PGx testing, statins, and SAMS, were estimated to be approximately 96 (Figure 3) to 104 USD (Table 3) greater for patients who received *SLCO1B1* results compared to those who did not for hypothetical patients and trial participants, respectively. This finding held after considering alternate assumptions for the cost of PGx testing, the cost of statin prescriptions, and the rates of simvastatin ordering. 

One possible reason for the limited effects observed in our trial-based analyses may be related to the limited number of study participants combined with overall low SAMS incidence rates [73], which are likely underreported in randomized trials and overreported in observational settings [74,75]. Even in a hypothetical cohort of 10,000 patients that were all prescribed simvastatin, we only projected 403 (4.0% of total patients on statins) total SAMS cases, of which approximately one-third (27.8%) of cases (109 myalgias and 3 myopathies) were estimated to have a genetic cause. Slightly higher SAMS incidence rates were observed in the I-PICC Study among statin users (10.0%, 5/50), with some cases reported by participants with a normal T/T genotype. Thus, while preemptive *SLCO1B1* testing may be able to prevent as many as one in three SAMS cases, it does not guarantee that SAMS will be avoided entirely [22]. 

Statin intensity, concomitant medication use, and demographic factors including ancestry, age, and sex have been associated with increased SAMS risk independent of *SLCO1B1* genotype [12,76]. Issues associated with potential nocebo effects [77] and health-related factors (e.g., other diagnoses and health status) and social factors may also play important roles in statin initiation and adherence more generally [78], further complicating the already challenging management of statin prescribing and intolerance [20]. Other important considerations that may impact the utility of *SLCO1B1* testing are recent changes in prescribing patterns and medication selection, such as the general transition away from simvastatin to atorvastatin as a first-option treatment [79,80]. On the other hand, this may eventually lead to additional reliance on *SLCO1B1* testing as a tool to help ameliorate SAMS-related events given mounting evidence of the relationships between *SLCO1B1* and both atorvastatin-related [10] and lovastatin-related [11] myotoxicity. However, due to the relative infrequency of SAMS, its multicausal nature, and the costs and outcomes observed here, standalone *SLCO1B1* testing may only be appropriate as a single test for patients predisposed to SAMS due to other risk factors or in a reactive manner for those who respond poorly to statin initiation [81,82].

A second perspective involves the inclusion of multiple well-validated pharmacogenes on a single panel [83]. Similar to Dong et al. [30] and Zhu et al. [31], our assessment demonstrated high initiation cost, driven mainly by the cost of PGx testing, that resulted in only nominal clinical benefits associated with *SLCO1B1*. Of note, cost effectiveness was not fully observed in these studies until a multigene pharmacogenomic panel (*SLCO1B1* included) was considered and outcomes were modeled over longer-term time horizons (24 months or longer). Importantly, the inclusion of *SLCO1B1* on these proposed multigene panels continued to offer some long-term incremental benefits to complement benefits provided by other well-validated pharmacogenes (e.g., *CYP2C19*-clopidogrel). When we modeled PGx testing at no cost to mirror a scenario where PGx results were already available in the medical record, we observed more manageable immediate expenses (PGx testing, lipid therapy prescriptions, and SAMS costs) across the full study sample (12.97 USD, 95% CI 1.34 to 25.09 USD; Table 4), and particularly among statin users with a C variant (*n* = 19, −9.94 USD, 95% CI −29.80 to 9.98 USD). In light of the existing literature, our findings are especially meaningful for the future of *SLCO1B1* testing given that well-developed multigene panels may be cost effective on the whole, despite some of their costlier, yet still potentially clinically actionable, individual parts [84].

### Limitations

Our study has a few notable limitations. The I-PICC Study was conducted at a single VHA health care system with its unique provider and patient populations, clinical practices, and electronic health record system. Thus, clinical outcomes associated with provider and patient behaviors and their downstream costs, including decisions about statin prescribing, how PGx information was made available to providers, and whether they used that information, may not be the same as other primary care settings. Implementation costs associated with educating providers, providing decision support, or overseeing program implementation were omitted. Additionally, our assessment focused solely on the health system perspective, with costs and outcomes derived nearly entirely from administrative databases. We did not consider patient costs that would inform analyses from the societal perspective, which was mainly due to a lack of availability of patient-level data and broader opportunity cost and outcome data. Our time horizon was limited to 12 months. This limitation was structural in that it mirrored the length of the I-PICC Study. This also impacted outcomes derived within our hypothetical cohort given the incorporation of the trial time frame and outcome data into our decision analytic modeling strategy. Further, given current CPIC recommendations, we only modeled simvastatin-related outcomes within our hypothetical cohort. Estimates here may be conservative with respect to the potential effects of *SLCO1B1* testing in light of mounting evidence for atorvastatin- and lovastatin-induced myotoxicity [10,11]. Future research assessing the costs and consequences of *SLCO1B1* testing may benefit from incorporating costs beyond those realized solely by the health system, as well as assessing costs and consequences across a range of statin medications, health care settings, and extended time horizons. 

## 5. Conclusions

Our analysis fills a unique gap in the literature as the first study to assess the cost implications of standalone and timely preemptive *SLCO1B1* testing in a primary care setting and provides initial evidence to inform future research. Though the mean per-patient costs to the health system were greater for PGx recipients, we observed minor clinical benefits at 1 month and 12 months in both our hypothetical veteran patient and I-PICC Study cohorts. Our findings align with those from other investigations and support the notion that, while *SLCO1B1* testing may be cost prohibitive as a single test, it may still have the potential to offer important incremental clinical benefits to primary care providers, at an acceptable cost threshold, when administered under specific conditions or included as part of a comprehensive multigene PGx testing strategy.

## Figures and Tables

**Table 1:** Characteristics of trial participants.

	PGx+ (*n* = 193)	PGx− (*n* = 215)	Total (*n* = 408)
Age at enrollment, mean (SD), years	64.2 (7.8)	63.9 (7.7)	64.1 (7.78)
Women, *n* (%)	9 (4.7)	16 (7.4)	25 (6.1)
Non-white race, *n* (%)	30 (15.5)	26 (12.1)	56 (13.7)
Hispanic ethnicity, *n* (%)	2 (1.0)	6 (2.8)	8 (2.0)
Smoker, *n* (%)	59 (30.1)	78 (36.3)	137 (33.6)
Baseline LDL-C, mean (SD), mg/dL	106 (32.0)	109 (28.0)	108 (30.0)
Meeting ACC/AHA statin criteria *, *n* (%)			
ASCVD	52 (26.9)	46 (21.4)	98 (24.0)
LDL-C > 190 mg/dL	5 (2.6)	6 (2.8)	11 (2.7)
Diabetes	47 (24.4)	51 (23.7)	98 (24.0)
10-year ASCVD risk ≥7.5%	171 (88.6)	196 (91.2)	367 (90.0)
*SLCO1B1* Genotype			
Reduced function T/C or C/C genotype, *n* (%)	45 (23.3)	75 (34.9)	120 (29.4)

* Categories sum greater to 100% because criteria are not mutually exclusive. Abbreviations: ACC/AHA, American College of Cardiology/American Heart Association; ASCVD, atherosclerotic cardiovascular disease; LDL-C, low-density lipoprotein cholesterol; mg/dL, milligram per deciliter; SD, standard deviation.

**Table 2:** Costs and consequences among I-PICC study participants over the 12-month trial period.

	Unadjusted Estimate		Adjusted Difference ^	95% CI	*p*
**12-month outcomes**	**PGx+ (*n* = 193)**	**PGx− (*n* = 215)**			
**Lipid prescriptions, *n* (%) USD**					
Offered statin	65 (33.7)	69 (32.1)	1.9%	−8.4%, 11.8%	0.687
Prescribed statin ~	26 (13.5)	24 (11.2)	2.9%	−4.2%, 9.0%	0.336
Atorvastatin *	19 (73.1)	19 (79.2)	−3.4%	−27.2%, 20.4%	0.779
Rosuvastatin *	0	3 (12.5)	−13.6%	−23.3%, −3.8%	0.006
Simvastatin *	7 (26.9)	2 (0.1)	17.1%	−3.3%, 37.6%	0.101
Provider-documented SAMS among statin users, *n* (%) *	2 (7.7)	3 (12.5)	−5.5%	−22.6%, 11.7%	0.533
Statin discontinuations *	3 (11.5)	4 (16.7)	−3.7%	−23.2, 15.8%	0.709
Other lipid medications	14 (7.3)	12 (5.6)	1.7%	−2.3%, 5.7%	0.421
**Utilization, mean (SD)**					
Inpatient stays	0.4 (1.3)	0.4 (1.4)	−0.1	−0.3, 0.2	0.694
Inpatient length of stay, days	4.8 (21.4)	4.5 (24.8)	0.3	−5.0, 4.9	0.892
Outpatient encounters, days	40.1 (31.0)	38.9 (27.2)	1.2	−4.1, 6.7	0.654
Primary care visits	3.9 (3.7)	4.7 (10.4)	−0.5	−3.8, 0.4	0.659
Cardiology visits	0.6 (1.3)	0.9 (1.9)	−0.2	−0.6, 0.1	0.131
**Costs, mean (SD), US dollars**					
Directly attributable costs	5648 (3122)	6407 (10,746)	−1004	−2684, 1009	0.284
SLCO1B1 PGx testing	99	0	99	−	
Lipid medications	17 (74)	8 (27)	10	−1, 23	0.140
Statins	6 (23)	4 (19)	2	−2, 6	0.291
Primary care	2955 (2802)	3400 (8950)	−445	−1414, 450	0.394
Cardiology	316 (901)	640 (2571)	−324	−915, 34	0.431
Imaging	1243 (2998)	1331 (3509)	−90	−701, 666	0.786
Laboratory	1015 (1574)	1027 (1386)	−11	−331, 366	0.946
SAMS-related care *	3 (27)	0 (0)	3	0, 5	0.045
Other outpatient services	8748 (15,263)	9381 (12,236)	−544	−3314, 3292	0.719
Physical inpatient stays	6107 (27,836)	4926 (18,887)	880	−4142, 6618	0.732
Total costs	20,497 (38,216)	20,706 (30,769)	−52	−6660, 8475	0.990

Utilization and cost data are summarized as mean per-patient estimates. Data presented here address both primary analyses of I-PICC participant data, which focus on directly attributable services (i.e., primary care and cardiology services, PGx testing, the cost of any lipid therapy prescription, and management of SAMS, including laboratory and imaging), and all observed costs and outcomes. ^ Generalized estimating equations (GEEs) were used to adjust mean differences, 95% confidence interval estimates, and *p*-values for clustering by provider. * Confidence interval and *p*-value derived from model-based standard error estimate. USD Per VA/DoD Guideline for Dyslipidemia Management [71,72]; ~ All 12-month statin prescriptions were concordant with Clinical Pharmacogenetics Implementation Consortium (CPIC) guidelines. Abbreviations: CI, confidence interval; PGx, pharmacogenetic; SAMS, statin-associated muscle symptoms; SD, standard deviation.

**Table 3:** Directly attributable mean per-patient costs for I-PICC participants over the 12-month trial period.

	Unadjusted Mean (SD), US Dollars		Adjusted Mean Difference and 95% CI ^	*p*
	**PGx+ (*n* = 193)**	**PGx− (*n* = 215)**		
PGx, Statins, SAMS	108 (39)	4 (19)	104 (97, 112)	0.001
PGx, Lipid Rx, SAMS	119 (80)	8 (27)	110 (98, 123)	<0.001
PGx, Lipid Rx, SAMS, Cardiology ^+^	434 (911)	649 (2573)	−215 (−710, 133)	0.307
PGx, Lipid Rx, SAMS, Primary Care ^+^	3074 (2810)	3409 (8952)	−335 (−1295, 518)	0.585
PGx, Lipid Rx, SAMS, Cardiology, Primary Care ^+^	3389 (3122)	4048 (9378)	−659 (−1687, 389)	0.243
PGx, Lipid Rx, SAMS, Cardiology, Primary Care, Laboratory, Imaging	5648 (5681)	6407 (10,746)	−1004 (−2684, 1009)	0.284

^ Generalized estimating equations (GEEs) used to adjust mean difference and 95% confidence interval estimates for clustering by provider. ^+^ Estimated using log-transformed dependent variable and heteroscedastic backtransformation. Abbreviations: CI, confidence interval; PGx, pharmacogenetic testing; SAMS, statin-associated muscle symptoms; SD, standard deviation.

**Table 4:** Scenario and sensitivity analyses: cost of PGx testing, cost of statin prescriptions, and statin initiations among C variant carriers over the 12-month trial period.

	Immediate Cost Mean Difference, 95% CI ^	Attributable Cost Mean Difference, 95% CI ^	Total Cost Mean Difference, 95% CI ^
**Base case, USD**	110 (98, 123) ***	−1004 (−2684, 1009)	−52 (−6660, 8475)
**Cost of PGx testing**			
No cost (−100%)	13 (1, 25) *	−1092 (−2714, 879)	−154 (−6761, 8371)
Lower bound (−25%)	85 (75, 97) **	−1026 (−2633, 949)	−78 (−6685, 8449)
Upper bound (+25%)	135 (123, 148) **	−981 (−2576, 952)	−26 (−6634, 8501)
**Cost of statin prescription**			
Lower bound (−50%)	109 (98, 120) **	−1004 (−2605, 1084)	−52 (−6659, 8475)
Upper bound (+200%)	112 (98, 126) **	−1003 (−2604, 1019)	−50 (−6661, 8475)
**Statin users**			
T/C or C/C genotype USD ~	74 (29, 119) **	4377 (−5061, 13,815)	3297 (−22,444, 29,039)

Immediate costs include PGx testing, lipid prescriptions, and SAMS costs. Attributable costs include immediate costs plus primary care, cardiology, imaging, and laboratory expenses. ^ Mean difference calculated as the mean cost in the intervention (PGx+) arm minus the mean cost in the usual care (PGx−) arm. ^ Generalized estimating equations (GEEs) used to correct mean difference and 95% confidence interval estimates for clustering by provider. USD estimate, confidence interval, and *p*-value derived from model-based standard error estimate. ~ 7/26 (26.9%) and 12/24 (50.0%) statin users in the PGx+ and PGx− arms, respectively, carried a C variant. Abbreviations: CI, confidence interval; PGx, pharmacogenetic. * *p* < 0.05, ** *p* < 0.01, *** *p* < 0.001.

## Data Availability

Deidentified participant data, Supplemental Materials, and analytic codes will be made available with investigator support after approval of the research proposal. Data will only be made available to researchers whose proposed use of the data has been approved for non-commercial purposes. Data request information can be found at: https://www.vacsp.research.va.gov/CSPEC/Studies/INVESTD-R/Integrating-Pharmacogenetics-clinical-care-study.asp (accessed on 8 October 2021).

## Supplementary Materials

The following are available online at https://www.mdpi.com/article/10.3390/jpm11111123/s1, Supplementary Materials and Methods, Table S1: Managerial Cost Accounting System (MCA) National Data Extracts inpatient Medicare-Severity Diagnosis Related Group (MS-DRG) codes excluded from inpatient health care costs, and stop codes used to derive total outpatient health care costs, Table S2: List of statin and non-statin pharmacologic agents included in study data extraction from the VA/DoD Clinical Practice Guideline for the Management of Dyslipidemia for Cardiovascular Risk Reduction, Table S3: International Classification of Disease (ICD) and Current Procedural Terminology (CPT) codes used for the identification of costs potentially associated with provider-documented SAMS, Table S4: Model inputs. Input parameters assume 100% initiation of simvastatin to provide insight about the maximum expected benefits from preemptive *SLCO1B1* genotyping (best-case scenario), Table S5: Results from analyses accounting for 12-month health care utilization and prior 12-month health care utilization and costs on 12-month lipid prescription costs, attributable costs, and total costs, Table S6: Best-case estimates and 95% CIs of 1-month muscle-related outcomes from modeling the best-case scenario, Table S7: Directly attributable and total costs among statin users and non-users over the 12-month trial period, Table S8: Total costs among the I-PICC Study participants over the 12-month trial period by high-risk subpopulation. Consolidated Health Economic Reporting Standards (CHEERS) checklist.
