# Supplementary material for: A Cost–Consequence Analysis of Preemptive SLCO1B1 Testing for Statin Myopathy Risk Compared to Usual Care
Source: J Pers Med. 2021 Oct 31;11(11):1123. doi: 10.3390/jpm11111123 (PMC8624003; doi:10.3390/jpm11111123)
Supplement: Supplementary file 1 [file jpm-11-01123-s001.zip › JPM_Supplement.pdf]

Supplementary Materials for

A cost-consequence analysis of preemptive *SLCO1B1* testing for statin myopathy risk compared to usual care

Charles A. Brunette, Olivia M. Dong, Jason L. Vassy, Morgan E. Danowski, Nicholas Alexander, Ashley A. Antwi, Kurt D. Christensen

Supplementary materials in this file include:

Methods and materials

Tables S1 to S8

References

Other Supplementary Materials for this manuscript include:

File S1. Consolidated Health Economic Reporting Standards (CHEERS) Checklist.

1. Methods and Materials

1.1. Statistical Analysis

Generalized estimating equations (GEE) have been consistently used within health economic settings to account for dependence among outcome data [1-5] and are amenable to the analysis of non-normally distributed data such as medical costs [6-11]. Moreover, GEEs have demonstrated appropriate performance in the estimation of treatment effects in cluster randomized trials with varying cluster size, when the number of clusters is sufficiently large (n>30), and within the context of pseudo-cluster randomization [12-15]. Per recommendations from Harden [16], parameters to calculate mean differences across arms were estimated using GEE and 95% cluster bootstrapped confidence intervals were calculated using resampling with replacement at the provider level [17,18]. *P*-values were calculated using the initial GEE parameter as reference to the bootstrapped distribution at the alpha 0.05 level (corresponding to a 95% confidence interval) [17]. In situations where sparse outcome data or small subsamples made it infeasible to perform cluster bootstrapping (e.g. SAMS), we used model-based standard error estimates to construct corresponding confidence intervals and *p*-values as recommended by Kahan et al. [15]. GEE bootstrapped estimates were comparable to those obtained using cluster bootstrapping with generalized linear models [19].

For the purposes of our primary analysis we included patient randomization status as the only covariate in our base GEE models given initial randomization at the provider level as well as unremarkable differences in observed patient characteristics across treatment arms [20] (**Table 1**) and clusters (e.g.

average cluster size) [21]. To further investigate the robustness of our findings for lipid medication, directly attributable, and total costs and to better parse potential cost drivers over and above randomization status alone, we conducted additional analyses including 12-month health care utilization (inpatient stays and outpatient encounter days) and prior 12-month health care utilization and costs as model covariates (**Supplementary Table S5**).

Table S1. Managerial Cost Accounting System (MCA) National Data Extracts inpatient Medicare-Severity Diagnosis Related Group (MS-DRG) codes excluded from inpatient health care costs and stop codes used to derive total outpatient health care costs.

**Inpatient care category**	**Excluded MS-DRG codes [22,23]**
Mental health, alcohol, and drug abuse and dependence care	876, 880, 881, 882, 883, 884, 885, 886, 887, 894, 895, 896, 897
**Outpatient care category**	**Included outpatient stop codes [24,25]**
Primary care	322, 323, 338
Cardiology	106, 107, 303, 311, 333, 334, 369, 391, 392
Medications (Lipid therapy prescriptions)	PHA, 160
Laboratory	108
Imaging	105, 109, 115, 150, 151, 153, 703
Home-based care	118, 171, 172, 173, 174, 175, 176, 178
Other general and specialty medical care<br><br>(Other general and specialty medical care does not include mental health care, social and welfare services, dental, optometry or visual services, speech pathology, or prosthetics costs)	103, 104, 116, 120, 123, 124, 130, 131, 139, 147, 148, 149, 182, 191, 197, 202, 205, 206, 210, 212, 214, 224, 225, 230, 241, 290, 291, 293, 301, 302, 304, 305, 307, 308, 309, 310, 312, 313, 314, 315, 316, 318, 321, 324, 325, 326, 328, 330, 336, 337, 340, 349, 350, 352, 353, 372, 373, 401, 402, 403, 404, 405, 406, 409, 410, 411, 413, 414, 415, 416, 419, 420, 421, 422, 424, 427, 429, 430, 432, 433, 434, 435, 529, 566, 571, 575, 593, 674, 683, 685, 999

Table S2. List of statin and non-statin pharmacologic agents included in study data extraction from the VA/DoD Clinical Practice Guideline for the Management of Dyslipidemia for Cardiovascular Risk Reduction (Versions 3* and 4) [26,27].

Drug category	Drug name
Statin	Atorvastatin, Fluvastatin, Lovastatin, Pitavastatin, Pravastatin, Rosuvastatin, Simvastatin
Cholesterol absorption inhibitors	Ezetimibe
PCSK9 inhibitors^	Alirocumab, Evolocumab
Fibrate	Fenofibrate, Fenofibric acid, Gemfibrozil
Bile acid sequestrant	Cholestyramine, Colestipol, Colesevelam
Niacin	Niaspan, Niacor
Fish oil	Fish oil

* Version 3.0 applicable during study period. ^Included from version 4.0.

Table S3. International Classification of Disease (ICD) and Current Procedural Terminology (CPT) codes used for the identification of costs potentially associated with provider-documented SAMS [28,29].

Disease state	ICD-9 code [30]	ICD-10 code [31]
Aminotransferase, elevated	790.4	R74.0
Creatinine clearance	794.4	R94.4
Electrolyte imbalance	276.9	E87.8
Hyperkalemia	276.7	E87.5
Hyperphosphatemia	275.3	E83.39
Hyperuricemia (asymptomatic)	790.6	E79.0
Hypocalcemia	275.41	E83.51
Hypovolemia	276.52	E86.1
Metabolic acidosis	276.2	E87.2
Myalgia (unspecified)	729.1	M79.10
Myoglobinuria	791.3	R82.1
Myopathy (drug induced)	359.4	G72.0
Myopathy (toxic)	359.4	G72.2
Myopathy (unspecified)	359.9	G72.9
Myositis (unspecified)	729.1	M60.9
Rhabdomyolysis (idiopathic)	728.88	M62.82
Elevated erythrocyte sedimentation rate	790.1	R70.0
Elevated C-Reactive Protein	790.95	R79.82
Aminotransferase, elevated	790.4	R74.0

Procedure	CPT code [32]
Creatinine Kinase	82550, 82552, 82553, 82554, 82565, 82570, 82575
Myoglobin	83874, 83885
Complete Blood Count	82025, 85027
Erythrocyte Sedimentation Rate (ESR)	85651, 85652
C-Reactive Protein	86141
Peripheral White Blood Cell (WBC) Count	85048
Potassium	84132, 84133
Phosphorous	84100, 84105
Calcium	82310, 82330, 82340
SGOT (aspartate aminotransferase)	84450
SGPT (alanine aminotransferase)	84460
LDH (lactic acid dehydrogenase)	83625

*Costs only considered if occurred in conjunction with provider-documented SAMS after date of documentation.

Table S4. Model inputs. Input parameters assume 100% initiation of simvastatin to provide insight about the maximum expected benefits from preemptive *SLCO1B1* genotyping (best-case scenario).

Input	Best-case value	Distribution (Range)	Reference
**Cohort size**	10,000		
**Receiving genetic testing**	10,000		
**SLCO1B1 variant carriers**	0.256	Triangle (0.233-0.349)	[33], IPICC study (low and high bounds)
**Statin therapy assignment**			
Proportion offered statin	1.0		Assumption
Proportion initiated statin	1.0		Assumption
Atorvastatin	0		Assumption
Rosuvastatin	0		Assumption
Simvastatin	1.0		Assumption
**1-month statin-related event probabilities**			
SLCO1B1-induced myalgia	0.0426	Beta (alpha: 182; beta: 4222)	[34]
Non-SLCO1B1 induced myalgia	0.0284	Beta (alpha: 122, beta: 4282)	[34]
Discontinue statin after myalgia	0.266	NA	[34]
SLCO1B1-induced myopathy	0.00111	Beta (alpha=11.1, beta=9989)	[34]

Non-SLCO1B1 induced myopathy	0.00074	Beta (alpha=7.4, beta=9992.6)	[34]
Discontinue statin after myopathy	0.403	NA	[34]
**Cost (2020 USD)**			
SLCO1B1 test	$99		IPICC study
Atorvastatin, 10-80 mg (per day)	$0.62	Triangle ($0.41,$0.62,$0.83)	[35]
Rosuvastatin, 5-40 mg (per day)	$0.69	Triangle ($0.52, $0.69, $0.87)	[35]
Simvastatin, 20-40 mg (per day)	$0.33	Triangle ($0.27, $0.33, $0.38)	[35]
Myopathy/myalgia management	$440	Triangle ($261, $440, $670)	[34]

Table S5. Results from analyses accounting for 12-month health care utilization and prior 12-month health care utilization and costs on 12-month lipid prescription costs, attributable costs, and total costs.

	**Prior and post 12-month adjusted costs and mean difference**				
	**PGx+ (n=193)**	**PGx- (n=215)**	**Mean difference**	**95% CI**	***p****
**Costs, mean (SD), US dollars**					
Prior 12-Month Lipid Prescription Costs	8.79 (37)	5.81 (31)	2.98	-4.01, 9.95	*0.404*
Post 12-Month Lipid Prescription Costs	17.90 (65)	8.40 (28)	9.53	-1.38, 22.38	*0.140*
Prior 12-Month Attributable Costs	5818 (7835)	5621 (5293)	197	-938, 1476	*0.745*
Post 12-Month Attributable Costs+	5684 (5724)	6688 (10,440)	-1004	-2684, 1009	*0.284*
Prior 12-Month Total Costs	22,790 (58,862)	18,327 (28,622)	4,536	-3097, 15,761	*0.289*
Post 12-Month Total Costs	21,502 (53,500)	21,554 (32,757)	-52	-6660, 8475	*0.990*

	**12-month adjusted costs and mean difference**				
	**PGx+ (n=193)**	**PGx- (n=215)**	**Mean difference**	**95% CI**	***p****
**Lipid Medication Costs**					
**12-Month Covariates**					
*Outpatient encounters^*	13.79 (51)	7.11 (19)	6.68	0.43, 18.20	0.032
**Prior 12-Month Covariates**					
*Prior 12-Month Lipid Prescription Costs^*	14.63 (31)	12.79 (27)	1.84	-2.29, 5.96	0.382
*Prior 12-Month Outpatient encounters^*<br>*Prior 12-Month Lipid Prescription Costs^*	14.71 (28)	13.37 (25)	1.34	-0.46, 3.15	0.145
**Attributable Costs**					
**12-Month Covariates**					
*Inpatient stays^*<br>*Outpatient encounters^*	4821 (3973)	4937 (3065)	-116	-775, 642	*0.751*
**Prior 12-Month Covariates**					
*Prior 12-Month Attributable Costs^*	5094 (3612)	5738 (6217)	-644	-1555, 459	*0.236*

*Prior 12-Month Outpatient encounters^*<br>*Prior 12-Month Attributable Costs^*	5006 (3584)	5731 (6672)	-725	-1645, 401	*0.190*
**Total Costs**					
**12-Month Covariates**					
*Inpatient stays^*<br>*Outpatient encounters^*	13,515 (14,823)	14,560 (10,924)	-1045	-3087, 1310	*0.374*
**Prior 12-Month Covariates**					
*Prior 12-Month Total Costs^*	16,625 (27,952)	18,994 (18,959)	-2369	-5774, 1899	*0.256*
*Prior 12-Month Outpatient encounters^*<br>*Prior 12-Month Total Costs^*	15,376 (21,533)	18,526 (18,417)	-3150	-5891, 185	*0.064*

Prior 12-month attributable costs include primary care, cardiology, imaging, laboratory, and lipid medication costs. Post 12-month attributable costs include primary care, cardiology, imaging, laboratory, lipid medication, SAMS, and PGx testing costs. Models included randomization status and additional covariates as described. Covariates were included or transformed if they provided better model fit via the QIC statistic [36]. Generalized Estimating Equations (GEEs) were used to correct within-group means, standard deviations, and mean differences for clustering. 95% confidence interval estimates and *p*-values were estimated using robust standard error estimates for models including covariates that did not converge when using cluster bootstrapped samples. **P*-value estimate for randomization status model coefficient. +Estimated using exchangeable correlation structure with log-transformed dependent variable. ^Model coefficients significant at $p < 0.05$. Abbreviations: CI, confidence interval; SD, standard deviation.

Table S6. Best-case estimates and 95% CIs of 1-month muscle-related outcomes from modeling the best-case scenario.

	**PGx testing**	**No PGx testing**
**Myalgia (*SLCO1B1*-induced)**	0 (NA)*	109 (94-141)
**Myalgia (non-*SLCO1B1*-induced)**	284 (231-325)	284 (231-325)
**Myopathy (*SLCO1B1*-induced)**	0 (NA)*	3 (2-5)
**Myopathy (non-*SLCO1B1*-induced)**	7 (5-10)	7 (5-10)

*Model assumes all patients with informative SLCO1B1 genotypes would not receive simvastatin therapy.

Table S7. Directly attributable and total costs among statin users and non-users over 12-month trial period.

	**Unadjusted mean (SD) costs, US dollars**		**Adjusted mean difference and 95% CI^**	*p*
**Statin Users (n=50)**	**PGx+ (n=26)**	**PGx- (n=24)**		
Statins	44 (49)	37 (45)	8 (-18, 34)	*0.560*
Lipid Rx	45 (49)	44 (50)	0 (-27, 27)	*0.979*
PGx, Statins, SAMS	164 (90)	37 (45)	127 (93, 171)	*0.001*
PGx, Lipid Rx, SAMS	164 (90)	44 (50)	121 (80, 165)	*0.001*
PGx, Lipid Rx, SAMS, Cardiology	679 (1182)	1139 (2113)	-498 (-1386, 563)	*0.312*
PGx, Lipid Rx, SAMS, Primary Care	3683 (2503)	4549 (3748)	-973 (-2693, 987)	*0.265*
PGx, Lipid Rx, SAMS, Cardiology, Primary Care	4198 (3061)	5644 (4823)	-1893 (-3659, 917)	*0.101*
PGx, Lipid Rx, SAMS, Cardiology, Primary Care, Laboratory, Imaging	7621 (9207)	7975 (7009)	-474 (-4303, 4484)	*0.834*
Total costs	20,842 (31,176)	28,914 (36,149)	-9225 (-27,150, 11,876)	0.346
**Non-users (n=358)**	**PGx+ (n=167)**	**PGx- (n=191)**		
Lipid Rx	13 (77)	4 (19)	9 (-1, 24)	*0.156*
PGx, Lipid Rx	112 (77)	4 (19)	108 (96, 120)	*0.002*
PGx, Lipid Rx, Cardiology+	396 (859)	587 (2623)	-191 (-754, 144)	*0.400*
PGx, Lipid Rx, Primary Care+	2979 (2850)	3265 (9400)	-286 (-1229, 660)	*0.611*
PGx, Lipid Rx, Cardiology, Primary Care+	3263 (3122)	3848 (9793)	-585 (-1627, 428)	*0.307*
PGx, Lipid Rx, SAMS, Cardiology, Primary Care, Laboratory, Imaging	5341 (4885)	6210 (11,125)	-1124 (-3028, 664)	*0.201*
Total costs	20,443 (39,278)	19,675 (29,976)	585 (-7519, 9014)	*0.868*

^Generalized Estimating Equations (GEEs) used to correct mean difference and 95% confidence interval estimates for clustering by provider. +Estimated using log-transformed dependent variable and heteroscedastic backtransformation. Abbreviations: CI, confidence interval; PGx, pharmacogenetic testing; SAMS, statin-associated muscle symptoms; SD, standard deviation.

Table S8. Total costs among I-PICC study participants over the 12-month trial period by high-risk subpopulation.

	Unadjusted mean (SD) costs, US dollars		Adjusted mean difference, 95% CI	95% CI	*p*
**High-risk subpopulation**	**PGx+**	**PGx-**			
ASCVD (n=98)	*20,823 (34,011)*	*21,046 (20,739)*	*-168*	*-9697, 13,684*	*0.972*
*LDL-C >= 190 (n=10)**	*16,532 (22,575)*	*12,622 (12,744)*	*-202*	*-33,073, 25,670*	*0.805*
Diabetes (n=77)	*21,829 (44,989)*	*23,934 (40,384)*	*-2950*	*-22,354, 19,983*	*0.769*
Risk >= 7.5% (n=223)	*20,098 (38,912)*	*19,729 (30,619)*	*131*	*-9397, 13,346*	*0.981*

Utilization and cost data are summarized as mean per-patient estimates. Data presented here address total observed costs for I-PICC Study participants. Generalized Estimating Equations (GEEs) were used to adjust mean differences, 95% confidence interval estimates, and *p*-values for clustering by provider. *Confidence interval and *p*-value derived from model-based standard error estimate. Abbreviations: ASCVD, atherosclerotic cardiovascular disease; CI, confidence interval; LDL-C, low-density lipoprotein cholesterol; SD, standard deviation.

Supplemental References

1. Gomes, M.; Grieve, R.; Nixon, R.; Edmunds, W.J. Statistical methods for cost-effectiveness analyses that use data from cluster randomized trials: a systematic review and checklist for critical appraisal. *Med Decis Making* **2012**, *32*, 209-220, doi:10.1177/0272989X11407341.
2. Gomes, M.; Ng, E.S.; Grieve, R.; Nixon, R.; Carpenter, J.; Thompson, S.G. Developing appropriate methods for cost-effectiveness analysis of cluster randomized trials. *Med Decis Making* **2012**, *32*, 350-361, doi:10.1177/0272989X11418372.
3. Athey, S.; Imbens, G.W. The Econometrics of Randomized Experiments. In *Handbook of Economic Field Experiments*, Banerjee, A.V., Duflo, E., Eds.; North-Holland: 2017; Volume 1, pp. 73-140.
4. Abadie, A.; Athey, S.; Imbens, G.W.; Wooldridge, J. When Should You Adjust Standard Errors for Clustering? *National Bureau of Economic Research Working Paper Series* **2017**, *No. 24003*, doi:10.3386/w24003.
5. El Alili, M.; van Dongen, J.M.; Goldfeld, K.S.; Heymans, M.W.; van Tulder, M.W.; Bosmans, J.E. Taking the Analysis of Trial-Based Economic Evaluations to the Next Level: The Importance of Accounting for Clustering. *Pharmacoeconomics* **2020**, *38*, 1247-1261, doi:10.1007/s40273-020-00946-y.
6. Titler, M.G.; Jensen, G.A.; Dochterman, J.M.; Xie, X.J.; Kanak, M.; Reed, D.; Shever, L.L. Cost of hospital care for older adults with heart failure: medical, pharmaceutical, and nursing costs. *Health Serv Res* **2008**, *43*, 635-655, doi:10.1111/j.1475-6773.2007.00789.x.
7. Larimer, M.E.; Malone, D.K.; Garner, M.D.; Atkins, D.C.; Burlingham, B.; Lonczak, H.S.; Tanzer, K.; Ginzler, J.; Clifasefi, S.L.; Hobson, W.G.; et al. Health care and public service use and costs before and after provision of housing for chronically homeless persons with severe alcohol problems. *JAMA* **2009**, *301*, 1349-1357, doi:10.1001/jama.2009.414.
8. Reed, S.D.; Li, Y.; Dunlap, M.E.; Kraus, W.E.; Samsa, G.P.; Schulman, K.A.; Zile, M.R.; Whellan, D.J. In-hospital resource use and medical costs in the last year of life by mode of death (from the HF-ACTION randomized controlled trial). *Am J Cardiol* **2012**, *110*, 1150-1155, doi:10.1016/j.amjcard.2012.05.059.
9. Paustian, M.L.; Alexander, J.A.; El Reda, D.K.; Wise, C.G.; Green, L.A.; Fetters, M.D. Partial and incremental PCMH practice transformation: implications for quality and costs. *Health Serv Res* **2014**, *49*, 52-74, doi:10.1111/1475-6773.12085.
10. Gidwani-Marszowski, R.; Owens, D.K.; Lo, J.; Goldhaber-Fiebert, J.D.; Asch, S.M.; Barnett, P.G. The Costs of Hepatitis C by Liver Disease Stage: Estimates from the Veterans Health Administration. *Appl Health Econ Health Policy* **2019**, *17*, 513-521, doi:10.1007/s40258-019-00468-5.
11. He, X.; Zhang, Y.; Zhou, Y.; Dong, C.; Wu, J. Direct Medical Costs of Incident Complications in Patients Newly Diagnosed With Type 2 Diabetes in China. *Diabetes Ther* **2021**, *12*, 275-288, doi:10.1007/s13300-020-00967-y.
12. Teerenstra, S.; Moerbeek, M.; Melis, R.J.; Borm, G.F. A comparison of methods to analyse continuous data from pseudo cluster randomized trials. *Stat Med* **2007**, *26*, 4100-4115, doi:10.1002/sim.2851.

13. Stedman, M.R.; Gagnon, D.R.; Lew, R.A.; Solomon, D.H.; Brookhart, M.A. An evaluation of statistical approaches for analyzing physician-randomized quality improvement interventions. *Contemp Clin Trials* **2008**, *29*, 687-695, doi:10.1016/j.cct.2008.04.003.
14. Chu, R.; Thabane, L.; Ma, J.; Holbrook, A.; Pullenayegum, E.; Devereaux, P.J. Comparing methods to estimate treatment effects on a continuous outcome in multicentre randomized controlled trials: a simulation study. *BMC Med Res Methodol* **2011**, *11*, 21, doi:10.1186/1471-2288-11-21.
15. Kahan, B.C.; Harhay, M.O. Many multicenter trials had few events per center, requiring analysis via random-effects models or GEEs. *J Clin Epidemiol* **2015**, *68*, 1504-1511, doi:10.1016/j.jclinepi.2015.03.016.
16. Harden, J.J. Improving Statistical Inference with Clustered Data. *Statistics, politics, and policy* **2012**, *3*, doi:10.2202/2151-7509.1026.
17. Davison, A.C.; Hinkley, D.V. *Bootstrap Methods and their Application*; Cambridge University Press: Cambridge, 1997.
18. Ren, S.; Lai, H.; Tong, W.; Aminzadeh, M.; Hou, X.; Lai, S. Nonparametric bootstrapping for hierarchical data. *Journal of Applied Statistics* **2010**, *37*, 1487-1498, doi:10.1080/02664760903046102.
19. Deen, M.; de Rooij, M. ClusterBootstrap: An R package for the analysis of hierarchical data using generalized linear models with the cluster bootstrap. *Behav Res Methods* **2020**, *52*, 572-590, doi:10.3758/s13428-019-01252-y.
20. Imai, K.; King, G.; Stuart, E.A. Misunderstandings between Experimentalists and Observationalists about Causal Inference. *Journal of the Royal Statistical Society. Series A, Statistics in society* **2008**, *171*, 481-502, doi:10.1111/j.1467-985X.2007.00527.x.
21. Gomes, M.; Grieve, R.; Nixon, R.; Ng, E.S.; Carpenter, J.; Thompson, S.G. Methods for covariate adjustment in cost-effectiveness analysis that use cluster randomised trials. *Health Econ* **2012**, *21*, 1101-1118, doi:10.1002/hec.2812.
22. Centers for Medicare and Medicaid Services. ICD-10-CM/PCS MS-DRG v39.0 Definitions Manual. Available online: https://www.cms.gov/icd10m/version38-fullcode-cms/fullcode_cms/P0002.html (accessed on June 3, 2020).
23. Wagner, T.H.; Chow, A.; Su, P.; Barnett, P.G. *HERC's Average Cost Datasets for VA Inpatient Care. Guidebook.*; VA Palo Alto, Health Economic Resource Center: Menlo Park, CA, May 2018
24. VA Information Resource Center. VIReC VHA Managerial Cost Accounting (MCA) Historical Stop Codes. . **2020**.
25. Phibbs, C.S.; Scott, W.J.; Flores, N.E.; Barnett, P.G. *HERC's Outpatient Average Cost Datasets for VA Care, FY1998-FY2018. Guidebook.*; VA Palo Alto, Health Economic Resource Center: Menlo Park, CA, June 2019.
26. The Management of Dyslipidemia for Cardiovascular Risk Reduction Work Group; Department of Veterans Affairs & Department of Defense. VA/DoD Clinical Practice Guideline for the Management of Dyslipidemia for Cardiovascular Risk Reduction (Version 3). Available online: https://www.healthquality.va.gov/guidelines/CD/lipids/VADoDDyslipidemiaCPG.pdf (accessed on August 4, 2020).
27. The Management of Dyslipidemia for Cardiovascular Risk Reduction Work Group; Department of Veterans Affairs & Department of Defense. VA/DoD Clinical Practice Guideline for the Management of Dyslipidemia for Cardiovascular Risk Reduction (Version 4). Available online: https://www.healthquality.va.gov/guidelines/CD/lipids/VADoDDyslipidemiaCPG5087212020.pdf (accessed on August 4, 2020).
28. Rosenson, R.S.; Baker, S.K. Statin muscle-related adverse events. Available online: https://www.uptodate.com/contents/statin-muscle-related-adverse-events (accessed on August 4, 2020).
29. Miller, M.L. Clinical manifestations and diagnosis of rhabdomyolysis. Available online: https://www.uptodate.com/contents/clinical-manifestations-and-diagnosis-of-rhabdomyolysis (accessed on August 4, 2020).
30. Centers for Medicare & Medicaid Services. International Classification of Disease 9 (ICD-9) Diagnosis and Procedure Codes. Available online: https://www.cms.gov/Medicare/Coding/ICD9ProviderDiagnosticCodes/codes (accessed on August 4, 2020).
31. Centers for Medicare & Medicaid Services. International Classification of Disease 10 (ICD-10). Available online: https://www.cms.gov/Medicare/Coding/ICD10/index (accessed on August 4, 2020).
32. American Medical Association. Current Procedural Terminology (CPT). Available online: https://www.ama-assn.org/amaone/cpt-current-procedural-terminology (accessed on August 4, 2020).
33. Chanfreau-Coffinier, C.; Hull, L.E.; Lynch, J.A.; DuVall, S.L.; Damrauer, S.M.; Cunningham, F.E.; Voight, B.F.; Matheny, M.E.; Oslin, D.W.; Icardi, M.S.; et al. Projected Prevalence of Actionable Pharmacogenetic Variants and Level A Drugs Prescribed Among US Veterans Health Administration Pharmacy Users. *JAMA Netw Open* **2019**, *2*, e195345, doi:10.1001/jamanetworkopen.2019.5345.

34. Dong, O.M.; Wheeler, S.B.; Cruden, G.; Lee, C.R.; Voora, D.; Dusetzina, S.B.; Wiltshire, T. Cost-Effectiveness of Multigene Pharmacogenetic Testing in Patients With Acute Coronary Syndrome After Percutaneous Coronary Intervention. *Value Health* **2020**, *23*, 61-73, doi:10.1016/j.jval.2019.08.002.
35. GoodRX.com. Available online: https://www.goodrx.com/ (accessed on March 26, 2021).
36. Pan, W. Akaike's information criterion in generalized estimating equations. *Biometrics* **2001**, *57*, 120-125, doi:10.1111/j.0006-341x.2001.00120.x.
